# Prosthetic Joint Infection: Updates on Prevention, Diagnosis and Therapy

**DOI:** 10.3390/jcm9123892

**Published:** 2020-11-30

**Authors:** Jiri Gallo

**Affiliations:** Department of Orthopedics, Faculty of Medicine and Dentistry, Palacky University Olomouc, University Hospital Olomouc, I. P. Pavlova 6, 77900 Olomouc, Czech Republic; jiri.gallo@volny.cz; Tel.: +420-58-8443548

Total joint arthroplasty (TJA) delivers highly valuable outcomes to patients with end-stage joint diseases. However, despite the investment in stratified preventative measurements, including the preoperative preparations of the patients, continuous improvements in the clinical settings, training of surgeons and operating room personnel, and perioperative antibiotics, prosthetic joint infection (PJI) remains the most frequent cause of early TJA failure. In addition, a projected increase in the use of TJAs will naturally result in a related increase in the number of PJIs. For this reason, further improvement of TJA practice is absolutely essential in order to control for the lowest incidence of PJI. This is not imaginable without commitment, continuous education, and tight monitoring of outcomes generated by each department providing joint arthroplasty care. This cannot be accomplished without quality improvement based on continuous data mining and the reflection of the efficacy of the preventative, diagnostic, and therapeutic strategy in all clinical settings.

A variety of host immune/non-immune cells interact perioperatively and early postoperatively with each other, contributing to the complex process of wound healing with an integration of the implant into the host tissues [1]. An integral part of these interactions is dedicated to clearing the peri-implant space from intraoperative contamination of the operative wound or an implant surface by bacteria. The first-line immune cells, including dendritic cells, macrophages, and neutrophils, recognise pathogen-associated molecular patterns (PAMPs) via a set of pattern recognition receptors (PRRs). These comprise a diverse set of proteins that are located on the cell surface or intracellularly [2]. A bacteria-related signal is translated into the expression of pro-inflammatory substances of anti-bacterial response via the stimulation of transcription gene modules. Two major innate immune defence responses triggered by PRRs are the type I interferon (IFN) and interleukin-1 (IL-1) mediated proinflammatory cascades [3]. Importantly, a number of effector cells migrate from blood vessels to the place of host–bacteria interaction to eradicate bacteria and subsequently resolve the anti-infective inflammation. A dysregulation of tightly regulated defence mechanisms can decrease or prevent the eradication of the intraoperative bacterial load. As a result, a bacterial biofilm can be established from constituent bacteria at the implant surface in several hours, which prevents immune cells from resolving infection.

This special issue of the Journal of Clinical Medicine is specifically dedicated to presenting current research topics on PJI. Two narrative reviews and six original articles are included.

Bozhkova and members of the World Association against Infection in Orthopaedics and Trauma (WAIOT) presented a study in which they validated previously [4] published criteria for PJI in this issue [5]. The validation process was conducted via a retrospective, multicentre, and international study of the proponents of the WAIOT definition of PJI. The authors discovered that the definition is a reliable tool for making diagnosis simple and accurate. However, the study has some limitations. One may suggest that the validation of any scientific work should be carried out by researchers from different institutions, but the same was carried out by other groups [6]. I feel that openness to criticism and an emphasis on the rigorous validation of any PJI definition is a crucial message.

Prevention is always better than treatment. In this issue, we published an extensive review, trying to overlook the field of PJI prevention [7]. It is difficult to comment on my own work, but let me express my strong belief that effective data collection and mining in conjunction with continuous quality improvement could be a critical strategy in the near future.

The identification of patients that are at risk preoperatively is part of any reasonable preventative strategy. This offers clinicians an opportunity to modify perioperative conditions in order to prevent PJI. Fujiwara et al. analysed the data of 121 patients undergoing the resection of musculoskeletal tumour of the lower limb conjoined with the implantation of tumour prosthesis [8]. This subgroup of patients is more prone to the development of PJI than patients without a local tumour at the time of TJA and there is a growing effort to stratify tumour patients individually according to additional risk factors. Using multivariate Cox proportional hazard models, the authors found that the risk of PJI may be associated with gender (male), the kind of tumour (extensive soft-tissue tumours), longer surgery, and local radiotherapy.

A critical step towards the prevention of PJI also lies in the detailed understanding of PJI development, from the initial step of bacterial adhesion to the building of a biofilm structure. Heim et al. examined synovial fluid and pre- and postsurgical blood samples to analyse the impact of TJA and spine surgery on peripheral blood leukocyte frequency, bactericidal activity, and the expression of inflammatory molecules [9]. We have known for a long time that the creation of biofilm on the implant surfaces is at least in part associated with the immunosuppressive effect of TJA [10]. This study reports an increased expression of IL-10, several growth factors, and an overall lack of pro-inflammatory mediators in the early postoperative phase. This only partly makes sense in relation to PJI, because periprosthetic tissues start with the activation of anti-inflammatory responses in a tight bond with surgery-induced inflammation. Thus, these findings tell us about a particular phase of the healing process rather than a failure of the inflammatory reaction that might be associated with the early development of PJI. Perhaps a more sophisticated study needs to be conducted with a series of samples taken at a specific time and with a wider spectrum of patients. However, the study is worth reading.

Two studies present data related to diagnostics of PJI. Enz et al. examined the diagnostic behaviour of minimally invasive biopsies in relation to the preoperative diagnosis of PJI [11]. Their data might support the implementation of a minimally invasive biopsy into the diagnostic algorithm for PJI, especially in cases with a dry puncture of a joint or negative synovial fluid culture in patients with an increased pretest probability of PJI. This intervention can be carried out in an ambulatory setting in a short time, with low risk of complications, and low cost. However, further studies must be conducted to support this method in the future.

Rüwald et al. examined the potential of extracellular vesicle (EV) isolation in the identification of PJI [12]. EVs are structures released into the environment by cells, including neutrophils, dendritic cells, and macrophages. The list of proteins that are specific to particular EV subtypes has been continuously expanding, similarly to our knowledge of their surface characteristics [13]. Importantly, these interesting structures have not been examined in relation to PJI to date. EVs were analysed in prosthetic pseudosynovial fluid obtained from patients with aseptic and septic TJAs using electron microscopy, nanoparticle tracking methodology, and a multiplex-bead-based extracellular EV analysis kit for 37 different EV surface markers and two isotype controls. The authors demonstrate the presence of EVs in both aseptic and septic pseudosynovial fluids. In addition, they show the differences between septic and aseptic cases in terms of the size, amount, and surface molecular structure of EVs. Samples from infected joints had higher EV concentrations and their EVs were smaller. Importantly, EVs produced as a part of an anti-infective response had different surface marker signatures compared to EVs from aseptic cases. Further investigation should clarify whether EV analysis should be included in the differential diagnosis of painful TJA.

The two final studies of this issue are related to the treatment of PJI. Firstly, Deroche et al. examined the optimal time for the safe re-evaluation of the empirical antibiotic choice during the operative treatment of hip and knee PJI [14]. The authors analysed data from 183 confirmed PJIs and concluded that almost 97% of all positive cultures were available within five days after the surgery when the culture protocol was optimised. An increase in cultivation time to seven days provided only a limited advantage in this study. This is in accordance with other studies that report a period within seven days as acceptable in the majority of PJIs [15]. However, some studies recommend waiting 10–14 days after taking multiple samples intraoperatively for the final culture outcomes [16]. These studies influenced our thinking, and those that have ended cultures before the seventh day have had to broadly explain their (mal)practice in meetings and in their publications. The International Consensus Meeting in Philadelphia (2018) recommended that “cultures should be maintained for a period of five to seven days, except cases of low-virulence organisms”, where two to three weeks were recommended [17]. However, it is not clear what “except cases of low-virulence” means clinically because when intraoperative samples are taken, no one knows whether the PJI is caused by low-virulence organisms. Perhaps, a low-grade infection may be a better indication.

Secondly, Kozaily et al. tried to examine a place for an additional spacer intervention (i.e., the initial antibiotic cement spacer is removed and a new spacer is inserted) during two-stage PJI treatment [18]. There may be mechanical and biological reasons for a spacer to be exchanged. Importantly, some of them may overlap at least in part with the reasons for a salvage procedure. Patients that are considered for cement spacer exchange typically have multiple comorbidities, chronic PJIs with resistant pathogens, previous spacer-related complications, or compromised soft tissues. The authors conclude that there is a place for spacer exchange in cases with persistent infection or mechanical complications. However, this practice should not result in the delay of a salvage intervention when it is justified. Further studies are required to improve our understanding of the situations of less responsive patients with PJI that would clarify the decision-making process.

This special issue is intended to offer the readers up-to-date and sound knowledge on a wide range of PJI topics. We believe that the articles have largely fulfilled these expectations. The editorial board pursued this project with the hope of contributing to new research to help tackle this increasingly prevalent and disabling complication of TJA. We would like to thank all of the authors and peer-reviewers for helping us with this excellent body of work.
